# Prevention of microbial hazard on fresh‐cut lettuce through adoption of food safety and hygienic practices by lettuce farmers

**DOI:** 10.1002/fsn3.365

**Published:** 2016-05-11

**Authors:** Lateefah A. Oyinlola, Adewale O. Obadina, Adebukunola M. Omemu, Olusola B. Oyewole

**Affiliations:** ^1^ Department of Food Science and TechnologyFederal University of AgricultureAbeokutaNigeria; ^2^ Department of Hospitality and Tourism ManagementFederal University of AgricultureAbeokutaNigeria

**Keywords:** Food safety, hygiene, lettuce, microbial contamination, pathogens

## Abstract

Lettuce is consumed raw in salads and is susceptible to microbial contamination through environment, agricultural practices, and its morphology, thus, a potential vehicle for food‐borne illness. This study investigated the effect of adoption of food safety and hygienic practices by lettuce farmers on the microbial safety of field sourced lettuce in Lagos State, Nigeria. Ten structured questionnaires were administered randomly to 10 lettuce farmers to assess food safety and hygienic practices (FSH). Two farmers who practice FSH and two farmers who do not practice NFSH were finally used for this study. Samples of ready‐to‐harvest lettuce, manure applied, and irrigation water were obtained for a period of five months (August – December 2013) and analyzed for total plate count (TPC), total coliform count (TCC), *Escherichia coli, Listeria spp., Salmonella spp.,* and *Shigella spp*. counts. Result of microbial analyses of lettuce samples was compared with international microbiological specification for ready‐to‐eat foods. Results showed that the range of TPC on lettuce was 6.00 to 8.11 LogCFU/g from FSH farms and TPC of lettuce samples from NFSH farms ranged from 6.66 to 13.64 LogCFU/g. 1.49 to 4.85LogCFU/g were TCC ranges from lettuce samples obtained from FSH farms while NFSH farms had TCC ranging between 3.95 and 10.86 LogCFU/g, respectively. The range of isolated pathogen count on lettuce from FSH and NFSH farms exceeded the international safety standard; there was a significant difference in the microbial count of lettuce from FSH farms and NFSH farms. This study concludes that the lettuce samples obtained did not pass the international microbial safety standards. FSH compliance is a major determinant of the microbial safety of lettuce. Hence, the institution of FSH on farm to improve microbial safety of lettuce produced for public consumption is emphasized.

## Introduction

Lettuce (*Lactuca sativa*) is a temperate annual or biennial plant of the daisy family Asteraceae (ITIS, 2010). It is often grown as a leafy vegetable eaten raw notably in salads, sandwiches, and hamburgers. Lettuce is healthy to eat because it is low in calories and contain essential nutrients (Gueye and Diouf 2007). Depending on the variety, lettuce is a good source of vitamin A, vitamin K, and Potassium with higher concentrations of vitamin A found in darker green lettuces. It also provides some dietary fiber (concentrated in the spine and ribs), carbohydrates, protein, and a small amount of fat with the exception of the iceberg type. Lettuce provides some vitamin C, calcium, iron, and copper, with vitamins and minerals largely found in the leaf (Katz and Weaver 2003). Lettuce is one of the vegetables that have attracted high consumption and have high economic importance throughout the world (FAOSTAT, 2010). It is a cool‐season crop that grows easily in a well‐drained, good soil rich in organic fertilizers with adequate supply of water. Water is the most dominant limiting factor for lettuce production (Coelho et al. 2005).

Human and animal enteric pathogens except soil‐borne spore formers such as *Clostridium perfringens* and *Bacillus cereus* are usually absent from fresh vegetables at harvest, unless they have been fertilized with human or animal wastes or irrigated with water containing such wastes (Generld et al. 2006). Raw vegetables and fruits may introduce pathogens into processing plants and kitchen environments, thus, contaminate other foods (Generld et al. 2006). Viruses, including hepatitis A, calicivirus, and Norwalk‐like strain have been found in lettuce (USFDA, 2012).

At production areas, irrigation and rinse waters have received attention, as they might be some of the major sources of microbial contamination. Irrigation and rinse waters might contain pathogenic bacteria such as *Salmonella* spp. and *Escherichia coli* O157:H7 (Generld et al. 2006). Usually, irrigation and rinse waters are used without any previous treatment when obtained from rivers, streams, lakes, or wells adjacent to the cropping areas (Pacheco et al. 2002; Abreu et al. 2010; Salem et al. 2011; Ilic et al. 2012; Olaimat and Holley 2012).

Food safety practices (FSH) are Good Agricultural Practices with bias for food safety on the farm. The concept of FSH evolved recently as a result of the big concern about food safety and quality, and the environmental sustainability of agriculture. FSH offers benefits to the farmers and consumers to meet specific objectives of food security, food quality, production efficiency, livelihood, and environmental protection. In a broad sense, FSH applies available knowledge in addressing environmental, economic, and social sustainability for on‐farm production and postproduction processing, resulting in a safe and healthy food and non‐food agricultural products.

A better understanding of the interaction between human pathogens and produce interaction is needed in the development of intervention strategies to increase the safety of lettuce supply. Thus, investigation on the microbial ecology of human pathogens, and factors affecting survival and growth of human pathogens in an agricultural/farm environment, including water and soil amendments, is essential to developing and implementation of the intervention to reduce the risk of contaminating fresh produce from the agricultural production environment.

## Materials and Methods

### Administration of questionnaires

Questionnaires were administered to 10 lettuce farmers within Badagry, Amuwo‐Odofin, and Ikorodu local governments of Lagos State, South Western Nigeria. The questionnaires were drafted to assess the food safety hygiene (FSH) practices of the farmers. Two farmers who practice (FSH) and two farmers who do not practice (NFSH) gave their consent to participate fully in the research work and thus used for this study. The different samples used for the analysis were collected from these farmers.

### Sample collection

Samples collected from the farms included lettuce, irrigation water, and Manure/soil samples.

### Sampling period

Sampling was done monthly between August and December 2013, which incorporated the weak rainy season and a weak dry season experienced which is an optimum period for lettuce growing in Lagos state. Sampling of lettuce, irrigation water, and soil around each sampled lettuce was done five times during the study period.

### Lettuce sampling

The lettuce plants were cut just above the ground with a knife previously disinfected with 70% ethyl alcohol. The samples were placed directly into sterile plastic bags. Samples were collected in replicate randomly from the farms during each visit.

### Irrigation water sampling

For the sampling of the irrigation water sources at each farm, 5 mL sample was collected in a sterilized plastic bottle. The sampling bottles were filled while turned sideways and upwards in order to avoid superficial contamination.

### Manure/soil sampling

Soil/manure samples were collected from a 10 cm^2^ area around each sampled lettuce plant. Soil samples were collected during every visit to the growers. At each production area, three soil samples were taken and pooled to produce one single soil sample for every grower on every visit. The samples were placed in plastic bags and transported to the laboratory for subsequent microbiological analyses.

### Microbiological analysis

All the samples (lettuce, irrigation water, manure/soil) were taken to the laboratory in an ice packed container for analysis. Nutrient agar (Macconkey agar, Salmonella–Shigella agar, Eosin ethylene blue agar and Brilliance Listeria agar) were used for total plate count (TPC), total coliform count (TCC), *Salmonella spp., Shigella spp*. *E. coli,* and *Listeria spp.,* counts, respectively, using standard bacteriological techniques as described by Eni et al. (2010). Isolates were identified using cultural, morphological, and biochemical characteristics. All media used were sourced from Oxoid, UK and prepared according to manufacturer's instructions. Microbial load on lettuce samples were compared with specification of “Guidelines for Assessing the Microbiological Safety of Ready‐to‐eat foods placed in Market” by “Health Protection Agency (2007)” and “Centre for Food Safety (2007)”, while irrigation water samples result was compared with the World Health Organization standards for irrigating fresh produce. (WHO, 1989, 2006)

### Data analysis

All procedures were carried out in triplicates and data collected from the study were subjected to analysis of variance (ANOVA). Differences among means were separated using Duncan's multiple range test and significances were accepted at 5% level (*P* < 0.05) (Duncan, 1955 ) Pearson correlation coefficient between the relative humidity, temperature, and rainfall of the sampling period as well as pathogenic load of manure, irrigation water, and lettuce samples was calculated. Statistical Package for Social Sciences 16.0 version was used for statistical.

## Results and discussion

The use of food safety and hygienic (FSH) practices among lettuce farmers was compared and summarized in Table 1. The farms visited received technical support, related to organic production practices, that was provided by extension services of the Lagos state ministry of agriculture, but the focus was mainly on the control of chemical hazards, example of which is pesticide residue, as this was derived from the interviews with the farmers. The workers were very compliant, responsive to changes, and concerned with possible quality improvements.

**Table 1:** Food safety hygiene (FSH) compliance by farmers. (*n* = 10)

Question on hygiene and food safety practices		Response (%)	
		Yes	No
1	Do you apply raw poultry droppings?	80	20
2	Do you apply raw cow dung?	70	30
3	Do you use human waste?	0	100
4	Is composted or aged animal manure used to supplement the soil?	100	0
5	If composted manure is used, are there records to show that animal manures are properly composted such as certifications or Standard Operating Procedures for composting?	0	100
6	Are there controls in place to prevent indirect contamination of raw animal manure from adjacent properties?	0	100
7	Do you keep untreated animal dungs with harvested vegetables?	0	100
8	Is the quality of water source assessed?	0	100
9	Do you use surface water for irrigation?	60	40
10	Do you use well water for irrigation?	40	60
11	Do you use pipe borne water for irrigation?	0	100
12	Do animals have access to irrigation water?	60	40
13	Do you store manure near source of irrigation?	30	70
14	Do you wash your hands before harvesting lettuce?	20	80
15	Do you wash lettuce vegetables after they are harvested?	0	100
16	Do you clean container for harvest before reusing them?	0	100
17	Do you use disinfectants to clean your harvest containers?	0	100
18	Do you have daily contact with animals (Poultry, Dogs, Goats, and Sheep)?	30	70
19	Do you wash your hands after touching animals?	0	100
20	Do birds or wildlife enter your vegetable farm?	60	40
21	Do you wash your hands after toilet use?	90	10
22	Do you wash your hands before eating?	80	20
23	Are you aware of anyone getting diarrhea from eating lettuce?	0	100
24	Is there a management program to identify	0	100
	potential contamination risks during the growing		
	and harvesting of lettuce?		

The majority of lettuce farmers (53%) applied raw poultry droppings to their lettuce plant, 70% applied raw cow dung, and none of the respondent made use of human waste. All (100%) the respondent agreed that they make use of composted or aged manure on lettuce farm. All the respondents had no control in place on the lettuce farms visited to prevent indirect contamination of raw animal manure from adjacent properties, while none of the respondents kept untreated animal dungs with harvested lettuce vegetables. The survey questionnaire result demonstrated that most of the lettuce farms operated in a high microbial risk context with respect to lettuce production, harvest, and packaging. Result also show that none (0%) of the respondents assessed the quality of the water, only 60% of the respondent uses surface water for irrigation, while 40% of the respondent used well water for irrigation and none (0%) of the respondent have access to tap water for irrigation purposes. Sixty percent (60%) of the respondent have birds or wildlife entering their farms and as such agreed that animals do have access to their source of irrigation water and majority (70%) of the respondent store their manure near source of irrigation water. Most (80%) of the respondent practice the hygiene of washing hands before harvesting lettuce vegetables, 90% claimed to wash their hands after using the toilet, 80% wash their hands before eating, and none of the respondents wash lettuce vegetables after they are harvested. None of the respondent uses disinfectant to clean harvest containers nor do they clean their containers before reusing them. Few (30%) of the respondents have daily contact with animals but none of the respondents washes their hand after touching animals.

Heaton and Jones (2008) reported that its a known and acceptable fact that fruit and vegetable consumption is a risk factor for infection of enteric pathogens, unfortunately, lettuce farmers interviewed lacked knowledge of lettuce contamination with food‐borne pathogens, and human illness. None of the respondents have been informed of anyone getting diarrheal infection from eating lettuce grown on their farm. Also, none (0%) of the respondents have a management program to identify potential contamination risk during the growing and harvesting of lettuce.

The farms visited received technical support, related to organic production practices, that was provided by extension services of the Lagos state ministry of agriculture, but the focus was mainly on the control of chemical hazards, example of which is the pesticide residue, as this was derived from the interviews with the farmers. The workers were very compliant, responsive to changes, and concerned with possible quality improvements.

On the average, TPC values from lettuce samples obtained from FSH 2 farm is the lowest, while TPC values from lettuce obtained from NFSH2 farm is the highest. A maximum acceptable concentration of 5.0 LogCFU/g of aerobic mesophylls on fresh‐cut vegetables is suggested by literature (Mossel 1982; Solberg et al. 1990) but TPC values obtained from this study ranged from 6.00 logCFU/g to 13.64 LogCFU/g. Munuera et al. (1994) also had lettuce samples with TPC values higher than 5.0 LogCFU/g in 61.5% of lettuce from different markets and street stands TPC values of 5.7, 7.8, 5.2, and 6.1 LogCFU/g were also reported for lettuce from salad bar, retail outlets, packaged vegetables, and grocery stores, respectively (Ercolani 1976; Garg et al. 1990; King et al. 1991; Albrecht et al. 1995).

The total coliform count (TCC) of lettuce samples obtained from FSH1 farm ranged from 4.77 LogCFU/g in November to 1.49 LogCFU/g in October, while FSH 2 farm had TCC values that ranged from 4.85 LogCFU/g in November to 3.78 LogCFU/g in December. TCC values of NFSH 1 farm ranged from 8.62 LogCFU/g in October to 3.95 LogCFU/g in December and TCC from NFSH 2 farm ranged from 10.86 LogCFU/g in November to 4.24 LogCFU/g in September. The mean TCC of lettuce samples obtained in each month of sampling were not significantly different from one another at *P* < 0.05, while the mean TCC values from different farms were significantly different *P* < 0.05 from one another as presented in Table 2. Total coliform count TCC on lettuce samples was at maximum with lettuce samples obtained from NFSH 2 (1.49 LogCFU/g) and was at minimum in FSH 1 lettuce samples (10.86 LogCFU/g) as presented in Table 2. Rodr′iguez de lecea and Soto Esteras (1981)while reporting 3.0 LogCFU/g as the standard TCC value expected on leafy greens TCC values found values lower than 3.0 LogCFU/g from his study, while Albrecht et al. (1995); Brocklehurst et al. (1987); Ercolani (1976) and Ruiz et al.(1987) found TCC higher values than 3.0 LogCFU/g. Due to their natural characteristics and the contact with soil, irrigation water, and animal intrusion, several studies have demonstrated that leafy greens frequently present contamination by fecal coliforms (Fischer‐Arndt et al. 2010; James 2006; Levantesi et al. 2012; Millner 2003; Moyne et al. 2011; Oliveira et al. 2012b;  and Oliveira et al. 2012b). In this study, there is neither 95% nor 99% correlation between TCC of lettuce and TCC of manure/soil samples as shown in Table 3.

**Table 2:** Microbial load of lettuce samples between August and December 2013

Months	Farm	TPC(Log CFU/g)	TCC(Log CFU/g)	*Shigella spp.Log CFU/25 g*	*Listeria spp.(Log CFU/25 g)*	*E. coli(Log CFU/25 g)*	*Salmonella spp.(Log CFU/25 g)*
August	FSH 1	7.24 ± 1.16_a_	4.47 ± 0.61_a_	2.30 ± 0.17_a_	4.30 ± 0.33_a_	2.00 ± 0.03_a_	2.00 ± 0.47_a_
	FSH 2	7.20 ± 0.96_ab_	4.64 ± 0.91_ab_	2.00 ± 0.55_a_	4.36 ± 0.65_a_	3.86 ± 0.67_a_	2.00 ± 0.48_a_
	NFSH	8.49 ± 1.72_c_	4.58 ± 0.25_c_	4.26 ± 0.33_a_	4.34 ± 0.97_a_	4.26 ± 0.65_a_	2.90 ± 0.53_a_
	NFSH 2	8.86 ± 1.52_bc_	8.14 ± 1.01_bc_	6.05 ± 1.16_a_	6.91 ± 1.16_a_	4.49 ± 0.81_a_	6.15 ± 0.50_a_
September	FSH 1	8.70 ± 1.20_a_	4.30 ± 0.60_a_	3.00 ± 0.27_a_	3.00 ± 0.95_a_	3.00 ± 0.30_a_	3.00 ± 0.11_a_
	FSH 2	7.00 ± 0.62_ab_	4.08 ± 0.55_ab_	3.00 ± 0.20_a_	3.48 ± 0.51_a_	3.00 ± 0.30_a_	3.00 ± 0.05_a_
	NFSH 1	7.20 ± 0.62_c_	4.86 ± 0.92_c_	3.47 ± 0.32_a_	5.70 ± 0.27_a_	3.00 ± 0.30_a_	3.00 ± 0.08_a_
	NFSH 2	7.70 ± 0.82_bc_	4.24 ± 1.23_bc_	3.18 ± 0.29_a_	8.30 ± 0.30_a_	3.65 ± 0.14_a_	1.69 ± 0.57_a_
October	FSH 1	7.26 ± 0.35_a_	1.49 ± 0.99_a_	4.34 ± 0.95_a_	9.08 ± 1.82_a_	0.00 ± 0.00_a_	3.00 ± 0.28_a_
	FSH 2	8.11 ± 1.13_ab_	4.20 ± 0.77_ab_	0.00 ± 0.00_a_	8.90 ± 1.20_a_	0.00 ± 0.00_a_	3.00 ± 0.28_a_
	NFSH 1	9.18 ± 0.79_c_	8.62 ± 1.65_c_	6.46 ± 0.82_a_	6.79 ± 1.00_a_	6.30 ± 0.91_a_	3.00 ± 0.28_a_
	NFSH 2	9.60 ± 1.69_bc_	6.62 ± 1.00_bc_	0.00 ± 0.00_a_	0.00 ± 0.00_a_	6.70 ± 0.90_a_	0.00 ± 0.00_a_
November	FSH1	6.26 ± 0.21_a_	4.77 ± 0.30_a_	4.56 ± 0.00_a_	5.70 ± 0.03_a_	3.00 ± 0.15_a_	3.00 ± 0.00_a_
	FSH 2	6.64 ± 0.32_ab_	4.85 ± 0.34_ab_	0.00 ± 0.00_a_	0.00 ± 0.00_a_	0.00 ± 0.00_a_	0.00 ± 0.00_a_
	NFSH 1	6.66 ± 0.32_c_	5.86 ± 0.78_c_	4.63 ± 0.00_a_	5.95 ± 0.02_a_	3.00 ± 0.15_a_	3.00 ± 0.00_a_
	NFSH 2	13.64 ± 0.31_bc_	10.86 ± 0.44_bc_	7.18 ± 0.00_a_	7.67 ± 0.05_a_	10.37 ± 0.30_a_	6.01 ± 0.00_a_
December	FSH 1	6.00 ± 0.22_a_	3.48 ± 0.19_a_	3.30 ± 0.05_a_	4.86 ± 0.20_a_	3.00 ± 0.06_a_	3.00 ± 0.00_a_
	FSH 2	6.18 ± 0.16_ab_	3.78 ± 0.24_ab_	3.30 ± 0.08_a_	4.69 ± 0.09_a_	3.00 ± 0.06_a_	3.00 ± 0.00_a_
	NFSH 1	6.93 ± 0.20_c_	3.95 ± 0.21_c_	3.48 ± 0.12_a_	4.75 ± 0.08_a_	3.00 ± 0.06_a_	3.00 ± 0.00_a_
	NFSH 2	8.53 ± 0.37_bc_	7.27 ± 0.32_bc_	4.41 ± 0.08_a_	2.30 ± 0.12_a_	5.45 ± 0.12_a_	3.01 ± 0.00_a_

Figures with the different subscript across columns shows significant difference (*P* < 0.05) in the mean of microbial result from different farms within a month, while figures with same subscripts across columns shows no significant difference (at *P *<* *0.05) from different farms within a month. FSH1, food safety hygiene compliance farm 1; FSH2, food safety hygiene compliance farm 2; NFSH1, food safety hygiene noncompliance farm 1, NFSH2, food safety hygiene noncompliance farm 2; TPC, total plate count; TCC, total coliform count.

**Table 3:** Correlation of total coliform counts in lettuce, manure, and irrigation water over season variation

TCC	Lettuce	Rainfall	Temperature	RH	Manure	Water
Lettuce	1.000					
Rainfall (mm)	0.180	1.000				
Temperature (°C)	−0.066	−0.207	1.000			
RH (%)	0.215	0.326	−0.458^b^	1.000		
Manure	0.349	−0.007	−0.065	0.115	1.000	
Water	0.523^a^	0.284	−0.048	0.099	−0.174	1.000

TCC, total coliform count; RH, relative humidity.

a Correlation is significant at the 0.01 level (two‐tailed).

b Correlation is significant at the 0.05 level (two‐tailed).

Mean *Shigella spp*. count, *E. coli* count, *Listeria spp*. count as well as *Salmonella spp*. count was at minimum from lettuce samples obtained from FSH 2 farm and peaked with lettuce samples obtained from NFSH 1 farm. *Listeria spp*. count obtained from lettuce samples throughout the research period exceeded the satisfactory limits of <1 LogCFU/g and border line of between 1 LogCFU/g and ≤2 LogCFU/g in Ready‐to‐eat vegetables. Chukwu et al.(2006) reported that outbreak of listeriosis has not yet been reported in Nigeria and there is limited report on the occurrence of the organism, this might be due to limited attention on the existence of the organism by public health workers and/or poor knowledge of its isolation and identification procedures. *E. coli* count on lettuce exceeded the satisfactory limit of “not detected in 25 g” reported by Health Protection Agency (2007) and Center for Food Safety (2007) throughout the research period. The presence of *E. coli* in vegetables may indicate insufficient awareness of microbial hazards during farming, inadequate sanitary conditions, and an increased probability of contamination by pathogenic bacteria associated with several food‐borne illnesses (Neto et al. 2012;  Soriano et al. 2000).

*Shigella spp*. count ranged between 8.07 LogCFU/25 g in “October” and 2.19 LogCFU/25 g in “August”, which were beyond the safety limits of “not detected in 25 g” reported by Health Protection Agency (2007). It also exceeded the safety level stated by Microbiological Guidelines For Ready‐to‐eat food (2007) which is <1.30 LogCFU/g as satisfactory level and 1.30103 LogCFU/g to <2 LogCFU/g being acceptable limits, *Salmonella spp*. count obtained from lettuce samples from this study exceeded the satisfactory safety limits of “not detected in 25 g” stated by Health Protection Agency (2007) and Microbiological Guidelines For ready‐to‐eat Food (2007).

Irrigation water samples also did not meet minimum World Health Organization (<1 LogCFU/mL) criteria for irrigating fresh produce‐like lettuce (World Health Organization 1989; WORLD HEALTH ORGANIZATION 2006) as presented in Table 4, this finding was in agreement with the previous reports (Okafo et al. 2003; Chigor et al. 2010). A significant positive correlation of coliform count of irrigation water with the coliform count of lettuce (*r* = 0.523, *P* < 0.01) presented in Table 3 as well as positive correlation between *Listeria spp*. count on irrigation water and the *Listeria spp*. count on lettuce presented on Table 5 vegetables suggests irrigation water sources being a subject to lettuce contamination in this study. FSH 1 and 2 used ground water while NFSH 1 and NFSH 2 used pond. Fong et al. (2007) and Richardson et al. (2009) identified that the ground water can be contaminated with different kinds of enteric microorganisms, such as *E. coli, Salmonellaspp*., and *Campylobacter*. However, Richardson et al. (2009) highlighted that the ground water is generally accepted to be of better quality because the water is protected from contamination more than pond water. Irrigation water samples, collected from both the water sources (pond water) and ground water indicated contamination by *E. coli* throughout the study period. FSH 1 and FSH 2 used ground water pumped up from a dug well for drip irrigation, meanwhile NFSH 1 and NFSH 2 farms used pond water for irrigation with bowls, buckets, and watering cans. None of the farmers had control in place to prevent indirect contamination of raw animal manure from adjacent properties; the well water can be suspected to be contaminated through adjacent properties especially when the water table is close to the ground surface. The exact cause of higher coliform contamination from NFSH lettuce samples was not determined, but low water quality, cross contamination, NFSH, and unknown factors could be contributing factors. The lack of sanitary and sewage management facilities in the study area coupled with close interactions between people, livestock, water sources, and fresh produce, could result in high concentrations of enteric organisms in the environment as well as increase the risk of producing cross contamination.

**Table 4:** Microbiological load of irrigation water samples (LogCFU/mL) between August and December 2013

Months	Farms	TPC(Log CFU/g)	TCC(Log CFU/g)	*Shigella spp.(Log CFU/25 g)*	*Listeria spp.Log CFU/g*	*E. coliLogCFU/25 g*	*Salmonella spp.(LogCFU/25 g)*
August	FSH 1	1.60 ± 0.06_a_	1.11 ± 0.08_a_	0.00 ± 0.00_a_	0.00 ± 0.05_a_	0.90 ± 0.05_a_	1.00 ± 0.10_a_
	FSH 2	1.90 ± 0.07_b_	1.08 ± 0.05_b_	0.00 ± 0.00_b_	0.48 ± 0.09_b_	1.30 ± 0.08_b_	1.60 ± 0.08_a_
	NFSH 1	2.13 ± 0.07_c_	1.64 ± 0.10_b_	0.00 ± 0.00_b_	0.00 ± 0.06_b_	1.36 ± 0.10_c_	3.00 ± 0.07_b_
	NFSH 2	2.01 ± 0.06_d_	1.28 ± 0.07_c_	0.00 ± 0.00_c_	0.16 ± 0.0.08_c_	1.67 ± 0.14_d_	1.16 ± 0.12_c_
September	FSH 1	3.30 ± 0.13_a_	2.45 ± 0.08_a_	0.85 ± 0.19_a_	1.53 ± 000_a_	2.70 ± 0.12_a_	4.00 ± 0.41_a_
	FSH 2	2.30 ± 0.10_b_	2.42 ± 0.08_b_	1.40 ± 0.17_b_	1.91 ± 0.05_b_	3.00 ± 0.10_b_	2.42 ± 0.40_a_
	NFSH 1	2.85 ± 0.17_c_	2.95 ± 0.05_b_	2.30 ± 0.20_b_	1.71 ± 0.06_b_	2.09 ± 0.13_c_	0.00 ± 0.70_b_
	NFSH 2	2.83 ± 0.12_d_	2.62 ± 0.11_c_	1.50 ± 0.18_c_	1.73 ± 0.03_c_	3.21 ± 0.14_d_	2.14 ± 0.50_c_
October	FSH 1	2.70 ± 0.15_a_	2.33 ± 0.01_a_	0.00 ± 0.00_a_	1.41 ± 0.07_a_	1.95 ± 0.14_a_	2.22 ± 0.12_a_
	FSH 2	2.10 ± 0.12_b_	2.30 ± 0.09_b_	0.66 ± 0.19_b_	1.79 ± 0.05_b_	2.53 ± 0.16_b_	2.82 ± 0.27_a_
	NFSH 1	2.70 ± 0.20_c_	2.85 ± 0.10_b_	0.78 ± 0.12_b_	1.96 ± 0.08_b_	2.60 ± 0.15_c_	3.91 ± 0.23_b_
	NFSH 2	3.30 ± 0.13_d_	2.49 ± 0.11_c_	1.28 ± 0.24_c_	1.71 ± 0.08_c_	3.08 ± 0.11_d_	2.30 ± 0.37_c_
November	FSH 1	3.52 ± 0.16_a_	2.48 ± 0.05_a_	0.00 ± 0.00_a_	1.61 ± 0.18_a_	2.18 ± 0.11_a_	2.30 ± 0.12_a_
	FSH 2	2.30 ± 0.20_b_	2.60 ± 0.08_b_	1.49 ± 0.18_b_	2.70 ± 0.20_b_	2.78 ± 0.17_b_	2.96 ± 0.30_a_
	NFSH 1	3.18 ± 0.11_c_	3.00 ± 0.09_b_	1.00 ± 0.21_b_	2.90 ± 0.19_b_	2.85 ± 0.16_c_	4.10 ± 0.32_b_
	NFSH 2	3.00 ± 0.23_d_	2.68 ± 0.03_c_	0.83 ± 0.18_c_	2.39 ± 0.17_c_	3.28 ± 0.14_d_	2.48 ± 0.25_c_
December	FSH 1	2.21 ± 0.08_a_	1.81 ± 0.06_a_	0.00 ± 0.00_a_	0.00 ± 0.00_a_	1.45 ± 0.00_a_	1.70 ± 0.002_a_
	FSH 2	2.58 ± 0.10_b_	1.90 ± 0.08_b_	0.36 ± 0.10_b_	0.95 ± 0.30_b_	2.04 ± 0.01_b_	1.86 ± 0.07_a_
	NFSH 1	2.81 ± 0.11_c_	2.33 ± 0.07_b_	0.30 ± 0.09_b_	0.90 ± 0.35_b_	2.13 ± 0.0_c_	2.10 ± 0.06_b_
	NFSH 2	2.68 ± 0.02_d_	2.00 ± 0.05_c_	0.78 ± 0.12_c_	1.99 ± 0.34_c_	2.58 ± 0.02_d_	1.78 ± 0.05_c_

Figures with the different subscript across columns shows significant difference (*P* < 0.05) in the mean of microbial result from different farms within a month, while figures with same subscripts across columns shows no significant difference (at *P* < 0.05) from different farms within a month. FSH1, food safety hygiene compliance farm 1; FSH2‐ food safety hygiene compliance farm 2; NFSH1, food safety hygiene noncompliance farm 1; NFSH2, food safety hygiene noncompliance farm 2. TPC, total plate count; TCC, total coliform count.

**Table 5:** Correlation of *Listeria spp*. in lettuce, manure and irrigation water over season

*Listeria spp*.	Lettuce	Rainfall	Temperature	RH	Manure	Water
Lettuce	1.000					
Rainfall (mm)	0.087	1.000				
Temperature (°C)	−0.054	−0.207	1.000			
RH (%)	0.268	0.326	−0.459^a^	1.000		
Manure	−0.200	0.266	−0.082	0.085	1.000	
Water	0.377^a^	0.241	0.185	−0.017	−0.411^a^	1.000

RH, relative humidity.

a Correlation is significant at the 0.05 level (two‐tailed).

The use of raw manure in vegetables produce contamination has been reported by many authors (Kudva et al. 1998; Ingham et al. 2004; Lejeune and Christie 2004). Table 6 presented the manure contamination during the research period, although an insignificant positive correlation (*r* = 0.349) existed between the TCC of the manure and TCC on lettuce samples as shown in Table 3. Manure samples from the farms showed high contamination of *E. coli* and coliforms, suggesting that composting were not thorough if practiced at all. Storage, frequency of application, and methods of application of manure were derived from the producers' own experiences. Table 7 An insignificant correlation that existed between the pathogenic load on lettuce and the pathogenic load on manure samples which can be attributed to disinfection of the manure with synthetic fertilizers during the application of urea and NPK fertilizers by the lettuce farmers to increase plant yield because nitrogen and phosphorus are usually the most limiting nutrients in many soils in Africa (SUGE et al. 2011). Organic inputs alone may not meet the nutritional needs of crops because they contain a comparatively less quantity of nutrients compared to inorganic fertilizers, therefore, there is no need to integrate the two forms in order to achieve better crop yields (Frankenberger and Abdelmagid 1985). As a small, uncharged molecule, ammonia can cross the bacterial membrane and cause damage to the cell either by causing rapid alkalization of the cytoplasm or through a decrease in intracellular K^+^ concentration (Kadam and Boone 1996; Park and Diez‐Gonzalez 2003). Furthermore, the insignificant correlation can be associated with other sources of contamination as highlighted by Quadrous Rodrigues et al. (2014) that seedlings also may be contaminated, especially when they are not treated with chemicals or have not undergone heat treatments before use, as in the case of some investigated organic farms that was studied. It can be said that this observation is a result of FSH compliance that was identified in the course of this research. FDA 2008 highlighted growing conditions and agricultural practices used by specific grower are risk factors that contribute to microbial contamination of vegetables.

**Table 6:** Microbial load of manure/soil samples between August and December 2013

Months	Farm	TPC *(LogCFU/g)*	TCC *(LogCFU/g)*	*Shigella spp. (LogCFU/25 g)*	*Listeria spp.(LogCFU/g)*	*E. coli (LogCFU/25 g)*	*Salmonella spp.(LogCFU/25 g)*
August	FSH 1	11.19 ± 0.12_a_	6.79 ± 0.28_a_	6.51 ± 0.21_a_	8.70 ± 0.04_a_	9.23 ± 1.12_a_	3.30 ± 0.05_a_
	FSH 2	11.47 ± 0.08_b_	8.15 ± 0.30_b_	6.86 ± 0.17_b_	9.23 ± 0.10_bc_	3.00 ± 0.74_a_	1.94 ± 0.05_ab_
	NFSH	10.61 ± 0.09_b_	5.90 ± 0.29_bc_	5.30 ± 0.23_bc_	8.97 ± 0.08_c_	4.43 ± 1.00_b_	7.36 ± 0.07_bc_
	NFSH 2	11.08 ± 0.11_b_	6.96 ± 0.27_c_	6.22 ± 0.20_c_	8.94 ± 0.12_c_	5.54 ± 1.4_c_	0.00 ± 0.03_c_
September	FSH 1	11.23 ± 0.31_a_	8.12 ± 0.32_a_	7.83 ± 0.32_a_	8.91 ± 0.27_a_	10.56 ± 1.11_a_	3.30 ± 1.4_a_
	FSH 2	8.95 ± 0.33_b_	8.18 ± 0.31_b_	6.86 ± 0.28_b_	9.28 ± 0.30_bc_	4.95 ± 0.85_a_	0.00 ± 0.00_ab_
	NFSH 1	10.97 ± 0.27_b_	5.94 ± 0.28_bc_	5.30 ± 0.33_bc_	10.95 ± 0.33_c_	4.46 ± 0.90_b_	8.09 ± 2.20_bc_
	NFSH 2	10.38 ± 0.30_b_	6.84 ± 0.34_c_	6.67 ± 0.31_c_	9.70 ± 0.18_c_	6.68 ± 0.78_c_	2.81 ± 1.20_c_
October	FSH 1	10.38 ± 0.24_a_	8.00 ± 0.31_a_	7.71 ± 0.27_a_	7.04 ± 0.29_a_	10.43 ± 0.99_a_	3.30 ± 1.00_a_
	FSH 2	9.00 ± 0.20_b_	8.25 ± 0.33_b_	6.86 ± 0.32_b_	9.28 ± 0.40_bc_	5.02 ± 0.90_a_	0.00 ± 0.00_ab_
	NFSH 1	10.89 ± 0.22_b_	6.00 ± 0.27_c_	5.30 ± 0.31_bc_	8.80 ± 0.27_c_	4.52 ± 0.81_b_	7.45 ± 1.9_bc_
	NFSH 2	10.10 ± 0.30_b_	7.40 ± 0.31_c_	6.61 ± 0.31_c_	8.36 ± 20_c_	6.68 ± 0.60_c_	4.42 ± 1.06_c_
November	FSH1	10.59 ± 0.25_a_	9.48 ± 0.46_a_	7.90 ± 0.66_a_	10.10 ± 0.22_a_	8.30 ± 0.48_a_	3.30 ± 0.93_a_
	FSH 2	8.86 ± 0.27_b_	8.09 ± 0.44_b_	6.86 ± 0.64_b_	9.20 ± 0.20_bc_	4.86 ± 0.54_a_	0.00 ± 0.00_ab_
	NFSH 1	10.73 ± 0.30_b_	5.85 ± 0.48_bc_	3.00 ± 0.60_bc_	10.90 ± 0.15_c_	4.20 ± 0.42_b_	6.73 ± 1.11_bc_
	NFSH 2	10.06 ± 0.22_b_	6.27 ± 0.42_c_	5.92 ± 0.62_c_	10.06 ± 0.27_c_	5.76 ± 0.32_c_	4.29 ± 0.72_c_
December	FSH 1	9.88 ± 0.16_a_	8.86 ± 0.51_a_	0.00 ± 0.00_a_	9.30 ± 0.24_a_	7.70 ± 0.50_a_	0.00 ± 0.00_a_
	FSH 2	8.73 ± 0.03_b_	7.95 ± 0.44_b_	0.00 ± 0.00_b_	9.00 ± 0.18_bc_	4.72 ± 0.75_a_	0.00 ± 0.00_ab_
	NFSH 1	10.70 ± 0.49_b_	5.00 ± 0.52_bc_	0.00 ± 0.00_bc_	10.79 ± 0.30_c_	4.08 ± 25_b_	2.24 ± 3.88_bc_
	NFSH 2	9.77 ± 0.29_b_	9.39 ± 054_c_	0.00 ± 0.00_c_	9.67 ± 0.21_c_	5.50 ± 52_c_	0.00 ± 0.00 _c_

Figures with the different subscript across columns shows significant difference (*P* < 0.05) in the mean of microbial result from different farms within a month, while figures with same subscripts across columns shows no significant difference (at *P* < 0.05) from different farms within a month. FSH1, food safety hygiene compliance farm 1, FSH2‐ food safety hygiene compliance farm 2; NFSH1, food safety hygiene noncompliance farm 1, NFSH2‐ food safety hygiene noncompliance farm 2. TPC, total plate count; TCC, total coliform count.

**Table 7:** Correlation of Total Plate Counts *in* lettuce, manure and irrigation water over seasonal variation

TPC	Lettuce	Rainfall	Temperature	RH	Manure	Water
Lettuce	1.000					
Rainfall (mm)	0.015	1.000				
Temperature (°C)	0.161	−0.207	1.000			
RH (%)	−0.144	0.326	−0.459^a^	1.000		
Manure	−0.119	−0.361^a^	−0.131	0.185	1.000	
Water	0.303	0.164	−0.014	0.016	−0.337	1.000

TPC, total plate count; RH, relative humidity.

a Correlation is significant at the 0.05 level (two‐tailed).

## Conclusion

Except for TPC and TCC, other microbial loads assessed were not significantly different at (*P* < 0.05) from one another across the farms (FSH compliance and NFSH compliance farm). It is also imperative that farmers and handlers of vegetables from irrigated fields should be educated on the measures aimed at minimizing or eliminating microbial hazards. Farmer and retailer education programs that lead to increased awareness and a change in food safety practices by lettuce value chain players in Nigeria may be an effective way of reducing the magnitude and frequency of lettuce contamination. The need for consumer awareness must be emphasized because vegetables may be affected by both microbiological and chemical contamination, and for that reason, sanitization procedures should be used to avoid food‐borne illnesses.

## Conflict of Interest

None declared.
